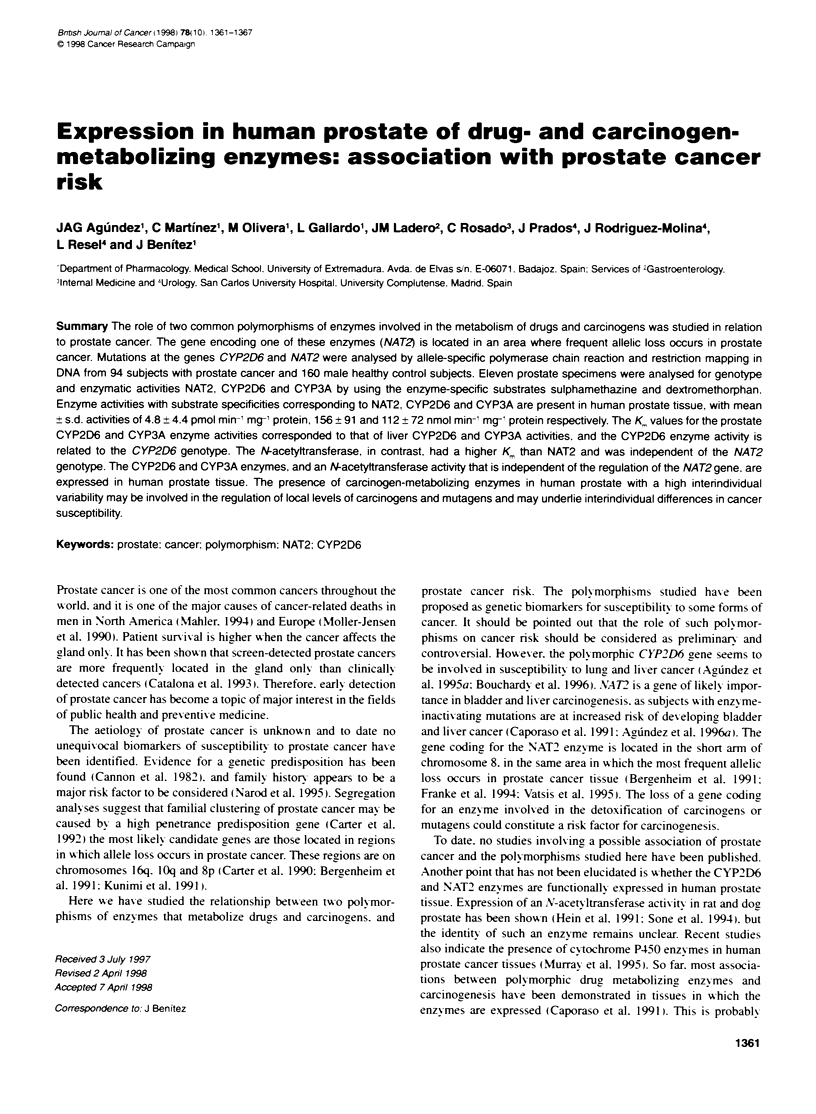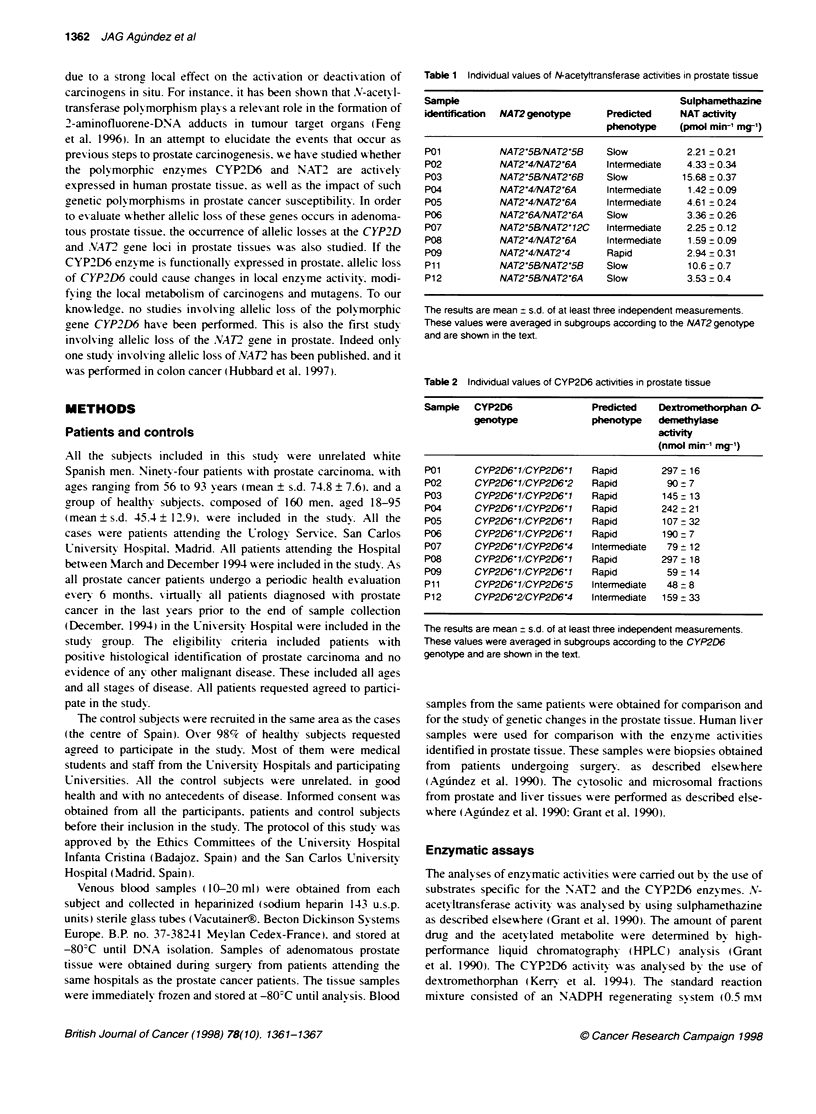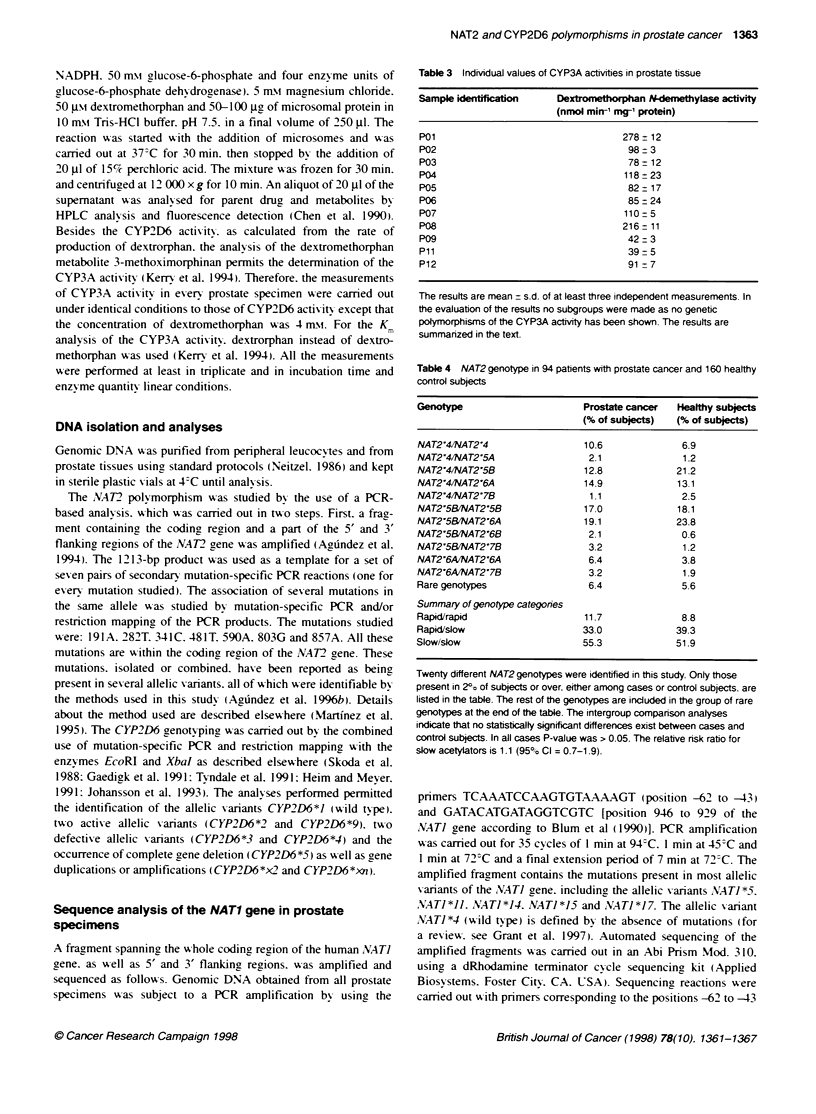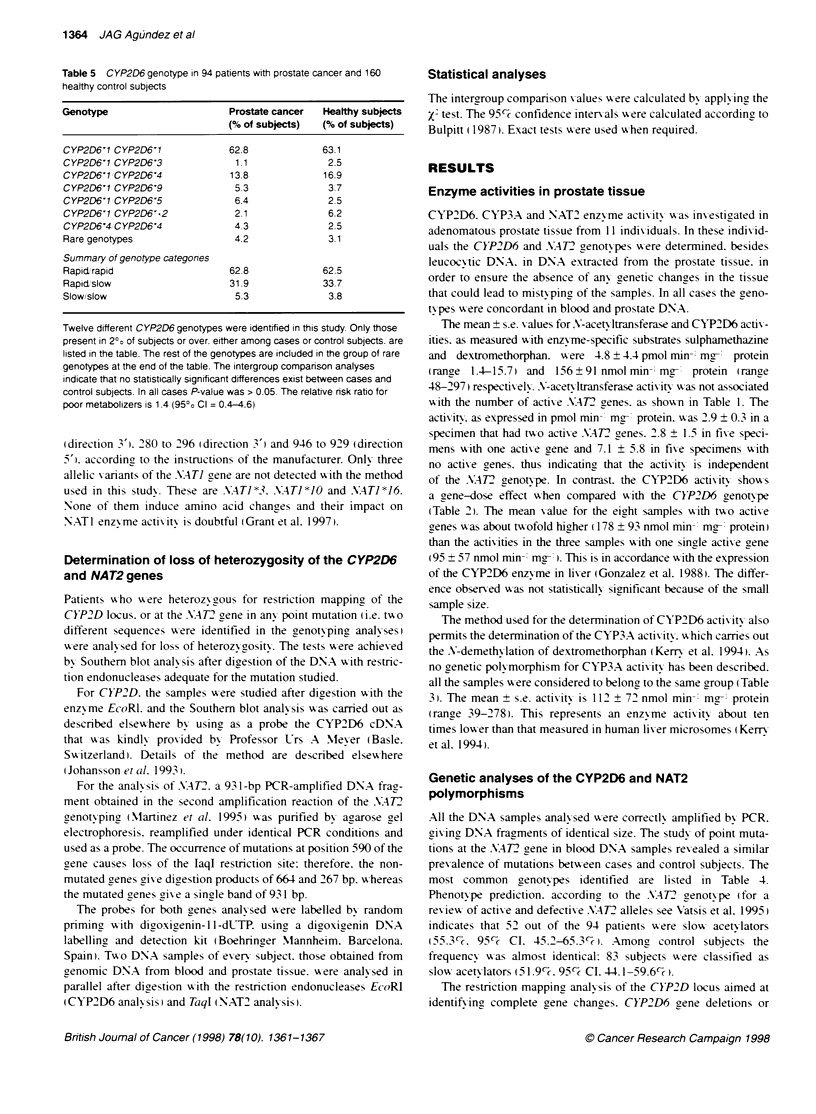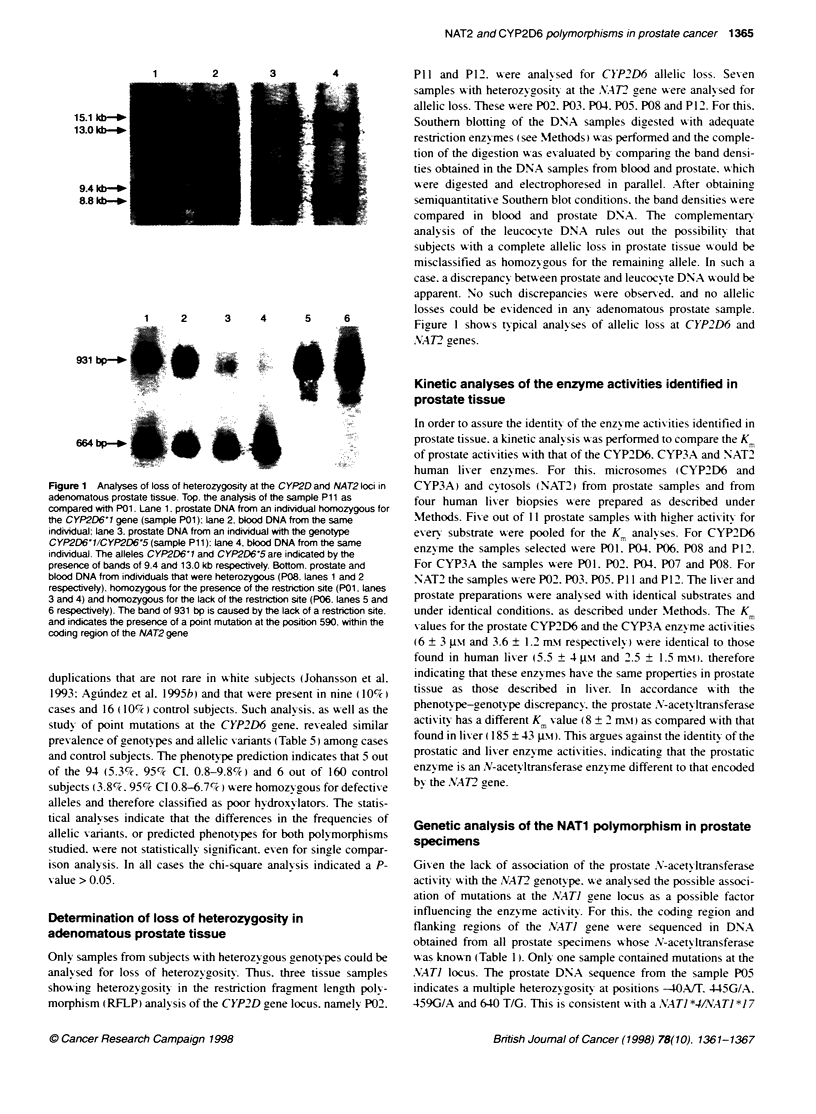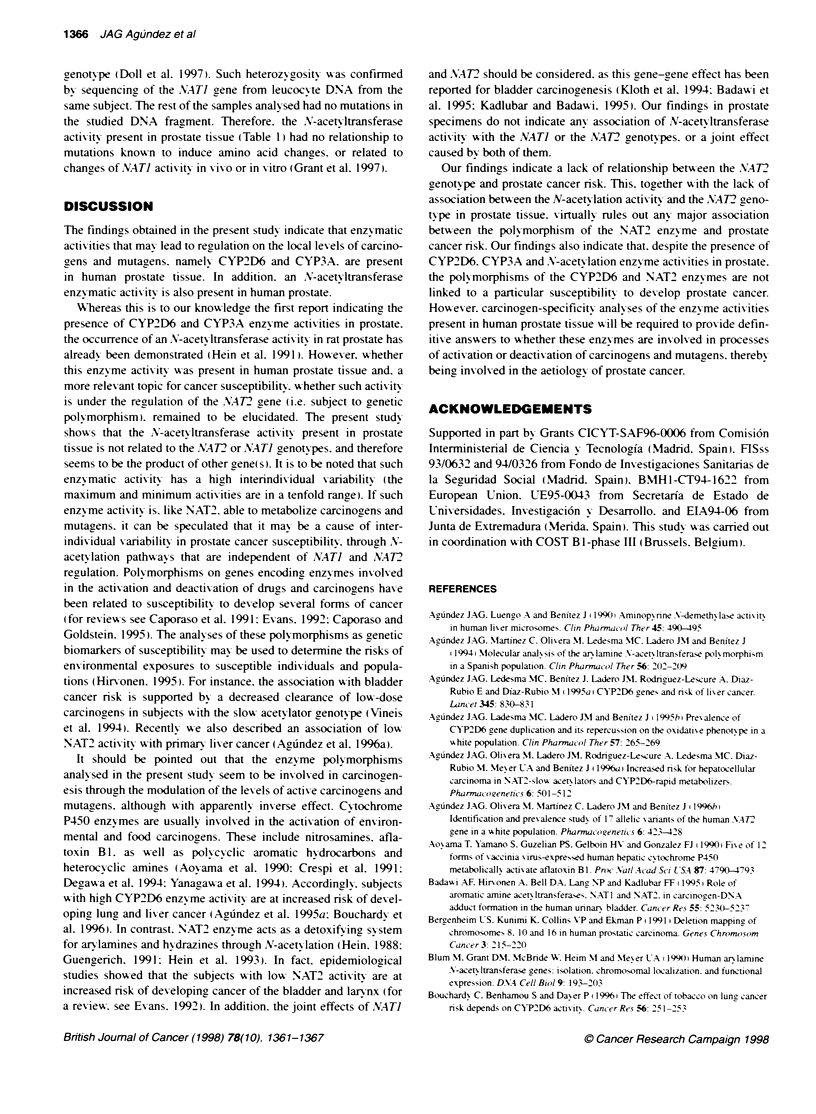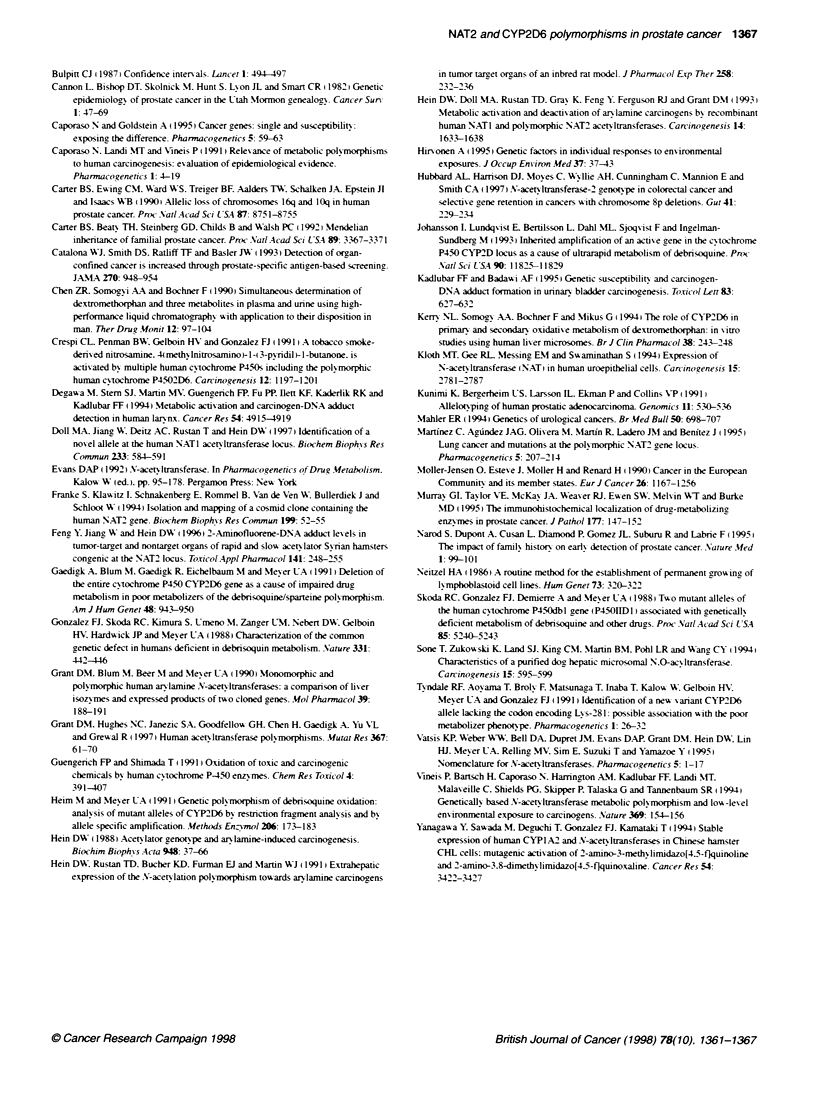# Expression in human prostate of drug- and carcinogen-metabolizing enzymes: association with prostate cancer risk.

**DOI:** 10.1038/bjc.1998.685

**Published:** 1998-11

**Authors:** J. A. Agúndez, C. Martínez, M. Olivera, L. Gallardo, J. M. Ladero, C. Rosado, J. Prados, J. Rodriguez-Molina, L. Resel, J. Benítez

**Affiliations:** Department of Pharmacology, Medical School, University of Extremadura, Badajoz, Spain.

## Abstract

**Images:**


British Journal of Cancer (1998) 78(10), 1361–1367
© 1998 Cancer Research Campaign

### Expression in human prostate of drug- and carcinogen-metabolizing enzymes: association with prostate cancer risk

**JAG Agúndez[1], C Martínez[1], M Olivera[1], L Gallardo[1], JM Ladero[2], C Rosado[3], J Prados[4], J Rodriguez-Molina[4], L Resel[4] and J Benítez[1]**

[1]Department of Pharmacology, Medical School, University of Extremadura, Avda. de Elvas s/n, E-06071, Badajoz, Spain; Services of [2]Gastroenterology, [3]Internal Medicine and [4]Urology, San Carlos University Hospital, University Complutense, Madrid, Spain

**Summary** The role of two common polymorphisms of enzymes involved in the metabolism of drugs and carcinogens was studied in relation to prostate cancer. The gene encoding one of these enzymes (*NAT2*) is located in an area where frequent allelic loss occurs in prostate cancer. Mutations at the genes *CYP2D6* and *NAT2* were analysed by allele-specific polymerase chain reaction and restriction mapping in DNA from 94 subjects with prostate cancer and 160 male healthy control subjects. Eleven prostate specimens were analysed for genotype and enzymatic activities NAT2, CYP2D6 and CYP3A by using the enzyme-specific substrates sulphamethazine and dextromethorphan. Enzyme activities with substrate specificities corresponding to NAT2, CYP2D6 and CYP3A are present in human prostate tissue, with mean ± s.d. activities of 4.8 ± 4.4 pmol $min^{-1}$ $mg^{-1}$ protein, 156 ± 91 and 112 ± 72 nmol $min^{-1}$ $mg^{-1}$ protein respectively. The $K_m$ values for the prostate CYP2D6 and CYP3A enzyme activities corresponded to that of liver CYP2D6 and CYP3A activities, and the CYP2D6 enzyme activity is related to the *CYP2D6* genotype. The *N*-acetyltransferase, in contrast, had a higher $K_m$ than NAT2 and was independent of the *NAT2* genotype. The CYP2D6 and CYP3A enzymes, and an *N*-acetyltransferase activity that is independent of the regulation of the *NAT2* gene, are expressed in human prostate tissue. The presence of carcinogen-metabolizing enzymes in human prostate with a high interindividual variability may be involved in the regulation of local levels of carcinogens and mutagens and may underlie interindividual differences in cancer susceptibility.

**Keywords:** prostate; cancer; polymorphism; NAT2; CYP2D6

Prostate cancer is one of the most common cancers throughout the world, and it is one of the major causes of cancer-related deaths in men in North America (Mahler, 1994) and Europe (Moller-Jensen et al, 1990). Patient survival is higher when the cancer affects the gland only. It has been shown that screen-detected prostate cancers are more frequently located in the gland only than clinically detected cancers (Catalona et al, 1993). Therefore, early detection of prostate cancer has become a topic of major interest in the fields of public health and preventive medicine.

The aetiology of prostate cancer is unknown and to date no unequivocal biomarkers of susceptibility to prostate cancer have been identified. Evidence for a genetic predisposition has been found (Cannon et al, 1982), and family history appears to be a major risk factor to be considered (Narod et al, 1995). Segregation analyses suggest that familial clustering of prostate cancer may be caused by a high penetrance predisposition gene (Carter et al, 1992) the most likely candidate genes are those located in regions in which allele loss occurs in prostate cancer. These regions are on chromosomes 16q, 10q and 8p (Carter et al, 1990; Bergenheim et al, 1991; Kunimi et al, 1991).

Here we have studied the relationship between two polymorphisms of enzymes that metabolize drugs and carcinogens, and prostate cancer risk. The polymorphisms studied have been proposed as genetic biomarkers for susceptibility to some forms of cancer. It should be pointed out that the role of such polymorphisms on cancer risk should be considered as preliminary and controversial. However, the polymorphic *CYP2D6* gene seems to be involved in susceptibility to lung and liver cancer (Agúndez et al, 1995*a*; Bouchardy et al, 1996). *NAT2* is a gene of likely importance in bladder and liver carcinogenesis, as subjects with enzyme-inactivating mutations are at increased risk of developing bladder and liver cancer (Caporaso et al, 1991; Agúndez et al, 1996*a*). The gene coding for the NAT2 enzyme is located in the short arm of chromosome 8, in the same area in which the most frequent allelic loss occurs in prostate cancer tissue (Bergenheim et al, 1991; Franke et al, 1994; Vatsis et al, 1995). The loss of a gene coding for an enzyme involved in the detoxification of carcinogens or mutagens could constitute a risk factor for carcinogenesis.

To date, no studies involving a possible association of prostate cancer and the polymorphisms studied here have been published. Another point that has not been elucidated is whether the CYP2D6 and NAT2 enzymes are functionally expressed in human prostate tissue. Expression of an *N*-acetyltransferase activity in rat and dog prostate has been shown (Hein et al, 1991; Sone et al, 1994), but the identity of such an enzyme remains unclear. Recent studies also indicate the presence of cytochrome P450 enzymes in human prostate cancer tissues (Murray et al, 1995). So far, most associations between polymorphic drug metabolizing enzymes and carcinogenesis have been demonstrated in tissues in which the enzymes are expressed (Caporaso et al, 1991). This is probably

*Received 3 July 1997*
*Revised 2 April 1998*
*Accepted 7 April 1998*

*Correspondence to:* J Benítez due to a strong local effect on the activation or deactivation of carcinogens in situ. For instance, it has been shown that N-acetyltransferase polymorphism plays a relevant role in the formation of 2-aminofluorene-DNA adducts in tumour target organs (Feng et al. 1996). In an attempt to elucidate the events that occur as previous steps to prostate carcinogenesis, we have studied whether the polymorphic enzymes CYP2D6 and NAT2 are actively expressed in human prostate tissue, as well as the impact of such genetic polymorphisms in prostate cancer susceptibility. In order to evaluate whether allelic loss of these genes occurs in adenomatous prostate tissue, the occurrence of allelic losses at the *CYP2D* and *NAT2* gene loci in prostate tissues was also studied. If the CYP2D6 enzyme is functionally expressed in prostate, allelic loss of *CYP2D6* could cause changes in local enzyme activity, modifying the local metabolism of carcinogens and mutagens. To our knowledge, no studies involving allelic loss of the polymorphic gene *CYP2D6* have been performed. This is also the first study involving allelic loss of the *NAT2* gene in prostate. Indeed only one study involving allelic loss of *NAT2* has been published, and it was performed in colon cancer (Hubbard et al. 1997).

### METHODS

### Patients and controls

All the subjects included in this study were unrelated white Spanish men. Ninety-four patients with prostate carcinoma, with ages ranging from 56 to 93 years (mean ± s.d. 74.8 ± 7.6), and a group of healthy subjects, composed of 160 men, aged 18–95 (mean ± s.d. 45.4 ± 12.9), were included in the study. All the cases were patients attending the Urology Service, San Carlos University Hospital, Madrid. All patients attending the Hospital between March and December 1994 were included in the study. As all prostate cancer patients undergo a periodic health evaluation every 6 months, virtually all patients diagnosed with prostate cancer in the last years prior to the end of sample collection (December, 1994) in the University Hospital were included in the study group. The eligibility criteria included patients with positive histological identification of prostate carcinoma and no evidence of any other malignant disease. These included all ages and all stages of disease. All patients requested agreed to participate in the study.

The control subjects were recruited in the same area as the cases (the centre of Spain). Over 98% of healthy subjects requested agreed to participate in the study. Most of them were medical students and staff from the University Hospitals and participating Universities. All the control subjects were unrelated, in good health and with no antecedents of disease. Informed consent was obtained from all the participants, patients and control subjects before their inclusion in the study. The protocol of this study was approved by the Ethics Committees of the University Hospital Infanta Cristina (Badajoz, Spain) and the San Carlos University Hospital (Madrid, Spain).

Venous blood samples (10–20 ml) were obtained from each subject and collected in heparinized (sodium heparin 143 u.s.p. units) sterile glass tubes (Vacutainer®, Becton Dickinson Systems Europe, B.P. no. 37-38241 Meylan Cedex-France), and stored at −80°C until DNA isolation. Samples of adenomatous prostate tissue were obtained during surgery from patients attending the same hospitals as the prostate cancer patients. The tissue samples were immediately frozen and stored at −80°C until analysis. Blood

**Table 1** Individual values of *N*-acetyltransferase activities in prostate tissue

| Sample identification | *NAT2* genotype | Predicted phenotype | Sulphamethazine NAT activity (pmol min$^{-1}$ mg$^{-1}$) |
|---|---|---|---|
| P01 | *NAT2*5B/NAT2*5B* | Slow | 2.21 ± 0.21 |
| P02 | *NAT2*4/NAT2*6A* | Intermediate | 4.33 ± 0.34 |
| P03 | *NAT2*5B/NAT2*6B* | Slow | 15.68 ± 0.37 |
| P04 | *NAT2*4/NAT2*6A* | Intermediate | 1.42 ± 0.09 |
| P05 | *NAT2*4/NAT2*6A* | Intermediate | 4.61 ± 0.24 |
| P06 | *NAT2*6A/NAT2*6A* | Slow | 3.36 ± 0.26 |
| P07 | *NAT2*5B/NAT2*12C* | Intermediate | 2.25 ± 0.12 |
| P08 | *NAT2*4/NAT2*6A* | Intermediate | 1.59 ± 0.09 |
| P09 | *NAT2*4/NAT2*4* | Rapid | 2.94 ± 0.31 |
| P11 | *NAT2*5B/NAT2*5B* | Slow | 10.6 ± 0.7 |
| P12 | *NAT2*5B/NAT2*6A* | Slow | 3.53 ± 0.4 |

The results are mean ± s.d. of at least three independent measurements. These values were averaged in subgroups according to the *NAT2* genotype and are shown in the text.

**Table 2** Individual values of CYP2D6 activities in prostate tissue

| Sample | CYP2D6 genotype | Predicted phenotype | Dextromethorphan *O*-demethylase activity (nmol min$^{-1}$ mg$^{-1}$) |
|---|---|---|---|
| P01 | *CYP2D6*1/CYP2D6*1* | Rapid | 297 ± 16 |
| P02 | *CYP2D6*1/CYP2D6*2* | Rapid | 90 ± 7 |
| P03 | *CYP2D6*1/CYP2D6*1* | Rapid | 145 ± 13 |
| P04 | *CYP2D6*1/CYP2D6*1* | Rapid | 242 ± 21 |
| P05 | *CYP2D6*1/CYP2D6*1* | Rapid | 107 ± 32 |
| P06 | *CYP2D6*1/CYP2D6*1* | Rapid | 190 ± 7 |
| P07 | *CYP2D6*1/CYP2D6*4* | Intermediate | 79 ± 12 |
| P08 | *CYP2D6*1/CYP2D6*1* | Rapid | 297 ± 18 |
| P09 | *CYP2D6*1/CYP2D6*1* | Rapid | 59 ± 14 |
| P11 | *CYP2D6*1/CYP2D6*5* | Intermediate | 48 ± 8 |
| P12 | *CYP2D6*2/CYP2D6*4* | Intermediate | 159 ± 33 |

The results are mean ± s.d. of at least three independent measurements. These values were averaged in subgroups according to the *CYP2D6* genotype and are shown in the text.

samples from the same patients were obtained for comparison and for the study of genetic changes in the prostate tissue. Human liver samples were used for comparison with the enzyme activities identified in prostate tissue. These samples were biopsies obtained from patients undergoing surgery, as described elsewhere (Agúndez et al. 1990). The cytosolic and microsomal fractions from prostate and liver tissues were performed as described elsewhere (Agúndez et al. 1990; Grant et al. 1990).

### Enzymatic assays

The analyses of enzymatic activities were carried out by the use of substrates specific for the NAT2 and the CYP2D6 enzymes. N-acetyltransferase activity was analysed by using sulphamethazine as described elsewhere (Grant et al. 1990). The amount of parent drug and the acetylated metabolite were determined by high-performance liquid chromatography (HPLC) analysis (Grant et al. 1990). The CYP2D6 activity was analysed by the use of dextromethorphan (Kerry et al. 1994). The standard reaction mixture consisted of an NADPH regenerating system (0.5 mM

 © Cancer Research Campaign 1998

NADPH, 50 mM glucose-6-phosphate and four enzyme units of glucose-6-phosphate dehydrogenase), 5 mM magnesium chloride, 50 μM dextromethorphan and 50–100 μg of microsomal protein in 10 mM Tris-HCl buffer, pH 7.5, in a final volume of 250 μl. The reaction was started with the addition of microsomes and was carried out at 37°C for 30 min, then stopped by the addition of 20 μl of 15% perchloric acid. The mixture was frozen for 30 min, and centrifuged at 12 000 × g for 10 min. An aliquot of 20 μl of the supernatant was analysed for parent drug and metabolites by HPLC analysis and fluorescence detection (Chen et al, 1990). Besides the CYP2D6 activity, as calculated from the rate of production of dextrorphan, the analysis of the dextromethorphan metabolite 3-methoximorphinan permits the determination of the CYP3A activity (Kerry et al, 1994). Therefore, the measurements of CYP3A activity in every prostate specimen were carried out under identical conditions to those of CYP2D6 activity except that the concentration of dextromethorphan was 4 mM. For the $K_m$ analysis of the CYP3A activity, dextrorphan instead of dextromethorphan was used (Kerry et al, 1994). All the measurements were performed at least in triplicate and in incubation time and enzyme quantity linear conditions.

### DNA isolation and analyses

Genomic DNA was purified from peripheral leucocytes and from prostate tissues using standard protocols (Neitzel, 1986) and kept in sterile plastic vials at 4°C until analysis.

The *NAT2* polymorphism was studied by the use of a PCR-based analysis, which was carried out in two steps. First, a fragment containing the coding region and a part of the 5′ and 3′ flanking regions of the *NAT2* gene was amplified (Agúndez et al, 1994). The 1213-bp product was used as a template for a set of seven pairs of secondary mutation-specific PCR reactions (one for every mutation studied). The association of several mutations in the same allele was studied by mutation-specific PCR and/or restriction mapping of the PCR products. The mutations studied were: 191A, 282T, 341C, 481T, 590A, 803G and 857A. All these mutations are within the coding region of the *NAT2* gene. These mutations, isolated or combined, have been reported as being present in several allelic variants, all of which were identifiable by the methods used in this study (Agúndez et al, 1996*b*). Details about the method used are described elsewhere (Martínez et al, 1995). The *CYP2D6* genotyping was carried out by the combined use of mutation-specific PCR and restriction mapping with the enzymes *Eco*RI and *XbaI* as described elsewhere (Skoda et al, 1988; Gaedigk et al, 1991; Tyndale et al, 1991; Heim and Meyer, 1991; Johansson et al, 1993). The analyses performed permitted the identification of the allelic variants *CYP2D6*1* (wild type), two active allelic variants (*CYP2D6*2* and *CYP2D6*9*), two defective allelic variants (*CYP2D6*3* and *CYP2D6*4*) and the occurrence of complete gene deletion (*CYP2D6*5*) as well as gene duplications or amplifications (*CYP2D6*x2* and *CYP2D6*xn*).

### Sequence analysis of the *NAT1* gene in prostate specimens

A fragment spanning the whole coding region of the human *NAT1* gene, as well as 5′ and 3′ flanking regions, was amplified and sequenced as follows. Genomic DNA obtained from all prostate specimens was subject to a PCR amplification by using the primers TCAAATCCAAGTGTAAAAGT (position –62 to –43) and GATACATGATAGGTCGTC [position 946 to 929 of the *NAT1* gene according to Blum et al (1990)]. PCR amplification was carried out for 35 cycles of 1 min at 94°C, 1 min at 45°C and 1 min at 72°C and a final extension period of 7 min at 72°C. The amplified fragment contains the mutations present in most allelic variants of the *NAT1* gene, including the allelic variants *NAT1*5*, *NAT1*11*, *NAT1*14*, *NAT1*15* and *NAT1*17*. The allelic variant *NAT1*4* (wild type) is defined by the absence of mutations (for a review, see Grant et al, 1997). Automated sequencing of the amplified fragments was carried out in an Abi Prism Mod. 310, using a dRhodamine terminator cycle sequencing kit (Applied Biosystems, Foster City, CA, USA). Sequencing reactions were carried out with primers corresponding to the positions –62 to –43

**Table 3** Individual values of CYP3A activities in prostate tissue

| Sample identification | Dextromethorphan *N*-demethylase activity (nmol min$^{-1}$ mg$^{-1}$ protein) |
|---|---|
| P01 | 278 ± 12 |
| P02 | 98 ± 3 |
| P03 | 78 ± 12 |
| P04 | 118 ± 23 |
| P05 | 82 ± 17 |
| P06 | 85 ± 24 |
| P07 | 110 ± 5 |
| P08 | 216 ± 11 |
| P09 | 42 ± 3 |
| P11 | 39 ± 5 |
| P12 | 91 ± 7 |

The results are mean ± s.d. of at least three independent measurements. In the evaluation of the results no subgroups were made as no genetic polymorphisms of the CYP3A activity has been shown. The results are summarized in the text.

**Table 4** *NAT2* genotype in 94 patients with prostate cancer and 160 healthy control subjects

| Genotype | Prostate cancer (% of subjects) | Healthy subjects (% of subjects) |
|---|---|---|
| *NAT2*4/NAT2*4* | 10.6 | 6.9 |
| *NAT2*4/NAT2*5A* | 2.1 | 1.2 |
| *NAT2*4/NAT2*5B* | 12.8 | 21.2 |
| *NAT2*4/NAT2*6A* | 14.9 | 13.1 |
| *NAT2*4/NAT2*7B* | 1.1 | 2.5 |
| *NAT2*5B/NAT2*5B* | 17.0 | 18.1 |
| *NAT2*5B/NAT2*6A* | 19.1 | 23.8 |
| *NAT2*5B/NAT2*6B* | 2.1 | 0.6 |
| *NAT2*5B/NAT2*7B* | 3.2 | 1.2 |
| *NAT2*6A/NAT2*6A* | 6.4 | 3.8 |
| *NAT2*6A/NAT2*7B* | 3.2 | 1.9 |
| Rare genotypes | 6.4 | 5.6 |
| *Summary of genotype categories* | | |
| Rapid/rapid | 11.7 | 8.8 |
| Rapid/slow | 33.0 | 39.3 |
| Slow/slow | 55.3 | 51.9 |

Twenty different *NAT2* genotypes were identified in this study. Only those present in 2% of subjects or over, either among cases or control subjects, are listed in the table. The rest of the genotypes are included in the group of rare genotypes at the end of the table. The intergroup comparison analyses indicate that no statistically significant differences exist between cases and control subjects. In all cases P-value was > 0.05. The relative risk ratio for slow acetylators is 1.1 (95% CI = 0.7–1.9).

© Cancer Research Campaign 1998

**Table 5** *CYP2D6* genotype in 94 patients with prostate cancer and 160 healthy control subjects

| Genotype | Prostate cancer (% of subjects) | Healthy subjects (% of subjects) |
|---|---|---|
| *CYP2D6*1/CYP2D6*1* | 62.8 | 63.1 |
| *CYP2D6*1/CYP2D6*3* | 1.1 | 2.5 |
| *CYP2D6*1/CYP2D6*4* | 13.8 | 16.9 |
| *CYP2D6*1/CYP2D6*9* | 5.3 | 3.7 |
| *CYP2D6*1/CYP2D6*5* | 6.4 | 2.5 |
| *CYP2D6*1/CYP2D6*×2* | 2.1 | 6.2 |
| *CYP2D6*4/CYP2D6*4* | 4.3 | 2.5 |
| Rare genotypes | 4.2 | 3.1 |
| *Summary of genotype categories* | | |
| Rapid/rapid | 62.8 | 62.5 |
| Rapid/slow | 31.9 | 33.7 |
| Slow/slow | 5.3 | 3.8 |

Twelve different *CYP2D6* genotypes were identified in this study. Only those present in 2% of subjects or over, either among cases or control subjects, are listed in the table. The rest of the genotypes are included in the group of rare genotypes at the end of the table. The intergroup comparison analyses indicate that no statistically significant differences exist between cases and control subjects. In all cases *P*-value was > 0.05. The relative risk ratio for poor metabolizers is 1.4 (95% CI = 0.4–4.6)

(direction 3′), 280 to 296 (direction 3′) and 946 to 929 (direction 5′), according to the instructions of the manufacturer. Only three allelic variants of the *NAT1* gene are not detected with the method used in this study. These are *NAT1*3*, *NAT1*10* and *NAT1*16*. None of them induce amino acid changes and their impact on NAT1 enzyme activity is doubtful (Grant et al, 1997).

### Determination of loss of heterozygosity of the *CYP2D6* and *NAT2* genes

Patients who were heterozygous for restriction mapping of the *CYP2D* locus, or at the *NAT2* gene in any point mutation (i.e. two different sequences were identified in the genotyping analyses) were analysed for loss of heterozygosity. The tests were achieved by Southern blot analysis after digestion of the DNA with restriction endonucleases adequate for the mutation studied.

For *CYP2D*, the samples were studied after digestion with the enzyme *Eco*RI, and the Southern blot analysis was carried out as described elsewhere by using as a probe the CYP2D6 cDNA that was kindly provided by Professor Urs A Meyer (Basle, Switzerland). Details of the method are described elsewhere (Johansson *et al*, 1993).

For the analysis of *NAT2*, a 931-bp PCR-amplified DNA fragment obtained in the second amplification reaction of the *NAT2* genotyping (Martinez *et al*, 1995) was purified by agarose gel electrophoresis, reamplified under identical PCR conditions and used as a probe. The occurrence of mutations at position 590 of the gene causes loss of the IaqI restriction site: therefore, the non-mutated genes give digestion products of 664 and 267 bp, whereas the mutated genes give a single band of 931 bp.

The probes for both genes analysed were labelled by random priming with digoxigenin-11-dUTP, using a digoxigenin DNA labelling and detection kit (Boehringer Mannheim, Barcelona, Spain). Two DNA samples of every subject, those obtained from genomic DNA from blood and prostate tissue, were analysed in parallel after digestion with the restriction endonucleases *Eco*RI (CYP2D6 analysis) and *Taq*I (NAT2 analysis).

### Statistical analyses

The intergroup comparison values were calculated by applying the $\chi^2$ test. The 95% confidence intervals were calculated according to Bulpitt (1987). Exact tests were used when required.

### RESULTS

### Enzyme activities in prostate tissue

CYP2D6, CYP3A and NAT2 enzyme activity was investigated in adenomatous prostate tissue from 11 individuals. In these individuals the *CYP2D6* and *NAT2* genotypes were determined, besides leucocytic DNA, in DNA extracted from the prostate tissue, in order to ensure the absence of any genetic changes in the tissue that could lead to mistyping of the samples. In all cases the genotypes were concordant in blood and prostate DNA.

The mean ± s.e. values for *N*-acetyltransferase and CYP2D6 activities, as measured with enzyme-specific substrates sulphamethazine and dextromethorphan, were 4.8 ± 4.4 pmol min$^{-1}$ mg$^{-1}$ protein (range 1.4–15.7) and 156 ± 91 nmol min$^{-1}$ mg$^{-1}$ protein (range 48–297) respectively. *N*-acetyltransferase activity was not associated with the number of active *NAT2* genes, as shown in Table 1. The activity, as expressed in pmol min$^{-1}$ mg$^{-1}$ protein, was 2.9 ± 0.3 in a specimen that had two active *NAT2* genes, 2.8 ± 1.5 in five specimens with one active gene and 7.1 ± 5.8 in five specimens with no active genes, thus indicating that the activity is independent of the *NAT2* genotype. In contrast, the CYP2D6 activity shows a gene–dose effect when compared with the *CYP2D6* genotype (Table 2). The mean value for the eight samples with two active genes was about twofold higher (178 ± 93 nmol min$^{-1}$ mg$^{-1}$ protein) than the activities in the three samples with one single active gene (95 ± 57 nmol min$^{-1}$ mg$^{-1}$). This is in accordance with the expression of the CYP2D6 enzyme in liver (Gonzalez et al, 1988). The difference observed was not statistically significant because of the small sample size.

The method used for the determination of CYP2D6 activity also permits the determination of the CYP3A activity, which carries out the *N*-demethylation of dextromethorphan (Kerry et al, 1994). As no genetic polymorphism for CYP3A activity has been described, all the samples were considered to belong to the same group (Table 3). The mean ± s.e. activity is 112 ± 72 nmol min$^{-1}$ mg$^{-1}$ protein (range 39–278). This represents an enzyme activity about ten times lower than that measured in human liver microsomes (Kerry et al, 1994).

### Genetic analyses of the CYP2D6 and NAT2 polymorphisms

All the DNA samples analysed were correctly amplified by PCR, giving DNA fragments of identical size. The study of point mutations at the *NAT2* gene in blood DNA samples revealed a similar prevalence of mutations between cases and control subjects. The most common genotypes identified are listed in Table 4. Phenotype prediction, according to the *NAT2* genotype (for a review of active and defective *NAT2* alleles see Vatsis et al, 1995) indicates that 52 out of the 94 patients were slow acetylators (55.3%, 95% CI, 45.2–65.3%). Among control subjects the frequency was almost identical: 83 subjects were classified as slow acetylators (51.9%, 95% CI, 44.1–59.6%).

The restriction mapping analysis of the *CYP2D* locus aimed at identifying complete gene changes, *CYP2D6* gene deletions or

 © Cancer Research Campaign 1998

**Figure 1** Analyses of loss of heterozygosity at the *CYP2D* and *NAT2* loci in adenomatous prostate tissue. Top, the analysis of the sample P11 as compared with P01. Lane 1, prostate DNA from an individual homozygous for the *CYP2D6*1* gene (sample P01); lane 2, blood DNA from the same individual; lane 3, prostate DNA from an individual with the genotype *CYP2D6*1/CYP2D6*5* (sample P11); lane 4, blood DNA from the same individual. The alleles *CYP2D6*1* and *CYP2D6*5* are indicated by the presence of bands of 9.4 and 13.0 kb respectively. Bottom, prostate and blood DNA from individuals that were heterozygous (P08, lanes 1 and 2 respectively), homozygous for the presence of the restriction site (P01, lanes 3 and 4) and homozygous for the lack of the restriction site (P06, lanes 5 and 6 respectively). The band of 931 bp is caused by the lack of a restriction site, and indicates the presence of a point mutation at the position 590, within the coding region of the *NAT2* gene duplications that are not rare in white subjects (Johansson et al, 1993; Agúndez et al, 1995*b*) and that were present in nine (10%) cases and 16 (10%) control subjects. Such analysis, as well as the study of point mutations at the *CYP2D6* gene, revealed similar prevalence of genotypes and allelic variants (Table 5) among cases and control subjects. The phenotype prediction indicates that 5 out of the 94 (5.3%, 95% CI, 0.8–9.8%) and 6 out of 160 control subjects (3.8%, 95% CI 0.8–6.7%) were homozygous for defective alleles and therefore classified as poor hydroxylators. The statistical analyses indicate that the differences in the frequencies of allelic variants, or predicted phenotypes for both polymorphisms studied, were not statistically significant, even for single comparison analysis. In all cases the chi-square analysis indicated a *P*-value > 0.05.

### Determination of loss of heterozygosity in adenomatous prostate tissue

Only samples from subjects with heterozygous genotypes could be analysed for loss of heterozygosity. Thus, three tissue samples showing heterozygosity in the restriction fragment length polymorphism (RFLP) analysis of the *CYP2D* gene locus, namely P02, P11 and P12, were analysed for *CYP2D6* allelic loss. Seven samples with heterozygosity at the *NAT2* gene were analysed for allelic loss. These were P02, P03, P04, P05, P08 and P12. For this, Southern blotting of the DNA samples digested with adequate restriction enzymes (see Methods) was performed and the completion of the digestion was evaluated by comparing the band densities obtained in the DNA samples from blood and prostate, which were digested and electrophoresed in parallel. After obtaining semiquantitative Southern blot conditions, the band densities were compared in blood and prostate DNA. The complementary analysis of the leucocyte DNA rules out the possibility that subjects with a complete allelic loss in prostate tissue would be misclassified as homozygous for the remaining allele. In such a case, a discrepancy between prostate and leucocyte DNA would be apparent. No such discrepancies were observed, and no allelic losses could be evidenced in any adenomatous prostate sample. Figure 1 shows typical analyses of allelic loss at *CYP2D6* and *NAT2* genes.

### Kinetic analyses of the enzyme activities identified in prostate tissue

In order to assure the identity of the enzyme activities identified in prostate tissue, a kinetic analysis was performed to compare the $K_m$ of prostate activities with that of the CYP2D6, CYP3A and NAT2 human liver enzymes. For this, microsomes (CYP2D6 and CYP3A) and cytosols (NAT2) from prostate samples and from four human liver biopsies were prepared as described under Methods. Five out of 11 prostate samples with higher activity for every substrate were pooled for the $K_m$ analyses. For CYP2D6 enzyme the samples selected were P01, P04, P06, P08 and P12. For CYP3A the samples were P01, P02, P04, P07 and P08. For NAT2 the samples were P02, P03, P05, P11 and P12. The liver and prostate preparations were analysed with identical substrates and under identical conditions, as described under Methods. The $K_m$ values for the prostate CYP2D6 and the CYP3A enzyme activities (6 ± 3 μM and 3.6 ± 1.2 mM respectively) were identical to those found in human liver (5.5 ± 4 μM and 2.5 ± 1.5 mM), therefore indicating that these enzymes have the same properties in prostate tissue as those described in liver. In accordance with the phenotype–genotype discrepancy, the prostate *N*-acetyltransferase activity has a different $K_m$ value (8 ± 2 mM) as compared with that found in liver (185 ± 43 μM). This argues against the identity of the prostatic and liver enzyme activities, indicating that the prostatic enzyme is an *N*-acetyltransferase enzyme different to that encoded by the *NAT2* gene.

### Genetic analysis of the NAT1 polymorphism in prostate specimens

Given the lack of association of the prostate *N*-acetyltransferase activity with the *NAT2* genotype, we analysed the possible association of mutations at the *NAT1* gene locus as a possible factor influencing the enzyme activity. For this, the coding region and flanking regions of the *NAT1* gene were sequenced in DNA obtained from all prostate specimens whose *N*-acetyltransferase was known (Table 1). Only one sample contained mutations at the *NAT1* locus. The prostate DNA sequence from the sample P05 indicates a multiple heterozygosity at positions –40A/T, 445G/A, 459G/A and 640 T/G. This is consistent with a *NAT1*4/NAT1*17*

© Cancer Research Campaign 1998

genotype (Doll et al. 1997). Such heterozygosity was confirmed by sequencing of the *NAT1* gene from leucocyte DNA from the same subject. The rest of the samples analysed had no mutations in the studied DNA fragment. Therefore, the *N*-acetyltransferase activity present in prostate tissue (Table 1) had no relationship to mutations known to induce amino acid changes, or related to changes of *NAT1* activity in vivo or in vitro (Grant et al. 1997).

### DISCUSSION

The findings obtained in the present study indicate that enzymatic activities that may lead to regulation on the local levels of carcinogens and mutagens, namely CYP2D6 and CYP3A, are present in human prostate tissue. In addition, an *N*-acetyltransferase enzymatic activity is also present in human prostate.

Whereas this is to our knowledge the first report indicating the presence of CYP2D6 and CYP3A enzyme activities in prostate, the occurrence of an *N*-acetyltransferase activity in rat prostate has already been demonstrated (Hein et al. 1991). However, whether this enzyme activity was present in human prostate tissue and, a more relevant topic for cancer susceptibility, whether such activity is under the regulation of the *NAT2* gene (i.e. subject to genetic polymorphism), remained to be elucidated. The present study shows that the *N*-acetyltransferase activity present in prostate tissue is not related to the *NAT2* or *NAT1* genotypes, and therefore seems to be the product of other gene(s). It is to be noted that such enzymatic activity has a high interindividual variability (the maximum and minimum activities are in a tenfold range). If such enzyme activity is, like NAT2, able to metabolize carcinogens and mutagens, it can be speculated that it may be a cause of inter-individual variability in prostate cancer susceptibility, through *N*-acetylation pathways that are independent of *NAT1* and *NAT2* regulation. Polymorphisms on genes encoding enzymes involved in the activation and deactivation of drugs and carcinogens have been related to susceptibility to develop several forms of cancer (for reviews see Caporaso et al. 1991; Evans, 1992; Caporaso and Goldstein, 1995). The analyses of these polymorphisms as genetic biomarkers of susceptibility may be used to determine the risks of environmental exposures to susceptible individuals and populations (Hirvonen, 1995). For instance, the association with bladder cancer risk is supported by a decreased clearance of low-dose carcinogens in subjects with the slow acetylator genotype (Vineis et al. 1994). Recently we also described an association of low NAT2 activity with primary liver cancer (Agúndez et al. 1996a).

It should be pointed out that the enzyme polymorphisms analysed in the present study seem to be involved in carcinogenesis through the modulation of the levels of active carcinogens and mutagens, although with apparently inverse effect. Cytochrome P450 enzymes are usually involved in the activation of environmental and food carcinogens. These include nitrosamines, aflatoxin B1, as well as polycyclic aromatic hydrocarbons and heterocyclic amines (Aoyama et al. 1990; Crespi et al. 1991; Degawa et al. 1994; Yanagawa et al. 1994). Accordingly, subjects with high CYP2D6 enzyme activity are at increased risk of developing lung and liver cancer (Agúndez et al. 1995*a*; Bouchardy et al. 1996). In contrast, NAT2 enzyme acts as a detoxifying system for arylamines and hydrazines through *N*-acetylation (Hein, 1988; Guengerich, 1991; Hein et al. 1993). In fact, epidemiological studies showed that the subjects with low NAT2 activity are at increased risk of developing cancer of the bladder and larynx (for a review, see Evans, 1992). In addition, the joint effects of *NAT1* and *NAT2* should be considered, as this gene–gene effect has been reported for bladder carcinogenesis (Kloth et al. 1994; Badawi et al. 1995; Kadlubar and Badawi, 1995). Our findings in prostate specimens do not indicate any association of *N*-acetyltransferase activity with the *NAT1* or the *NAT2* genotypes, or a joint effect caused by both of them.

Our findings indicate a lack of relationship between the *NAT2* genotype and prostate cancer risk. This, together with the lack of association between the *N*-acetylation activity and the *NAT2* genotype in prostate tissue, virtually rules out any major association between the polymorphism of the NAT2 enzyme and prostate cancer risk. Our findings also indicate that, despite the presence of CYP2D6, CYP3A and *N*-acetylation enzyme activities in prostate, the polymorphisms of the CYP2D6 and NAT2 enzymes are not linked to a particular susceptibility to develop prostate cancer. However, carcinogen-specificity analyses of the enzyme activities present in human prostate tissue will be required to provide definitive answers to whether these enzymes are involved in processes of activation or deactivation of carcinogens and mutagens, thereby being involved in the aetiology of prostate cancer.

### ACKNOWLEDGEMENTS

Supported in part by Grants CICYT-SAF96-0006 from Comisión Interministerial de Ciencia y Tecnología (Madrid, Spain), FISss 93/0632 and 94/0326 from Fondo de Investigaciones Sanitarias de la Seguridad Social (Madrid, Spain), BMH1-CT94-1622 from European Union, UE95-0043 from Secretaría de Estado de Universidades, Investigación y Desarrollo, and EIA94-06 from Junta de Extremadura (Merida, Spain). This study was carried out in coordination with COST B1-phase III (Brussels, Belgium).

### REFERENCES

Agúndez JAG, Luengo A and Benítez J (1990) Aminopyrine *N*-demethylase activity in human liver microsomes. *Clin Pharmacol Ther* **45**: 490–495

Agúndez JAG, Martínez C, Olivera M, Ledesma MC, Ladero JM and Benítez J (1994) Molecular analysis of the arylamine *N*-acetyltransferase polymorphism in a Spanish population. *Clin Pharmacol Ther* **56**: 202–209

Agúndez JAG, Ledesma MC, Benítez J, Ladero JM, Rodriguez-Lescure A, Diaz-Rubio E and Díaz-Rubio M (1995*a*) CYP2D6 genes and risk of liver cancer. *Lancet* **345**: 830–831

Agúndez JAG, Ladesma MC, Ladero JM and Benítez J (1995*b*) Prevalence of CYP2D6 gene duplication and its repercussion on the oxidative phenotype in a white population. *Clin Pharmacol Ther* **57**: 265–269

Agúndez JAG, Olivera M, Ladero JM, Rodriguez-Lescure A, Ledesma MC, Diaz-Rubio M, Meyer UA and Benítez J (1996*a*) Increased risk for hepatocellular carcinoma in NAT2-slow acetylators and CYP2D6-rapid metabolizers. *Pharmacogenetics* **6**: 501–512

Agúndez JAG, Olivera M, Martínez C, Ladero JM and Benítez J (1996*b*) Identification and prevalence study of 17 allelic variants of the human *NAT2* gene in a white population. *Pharmacogenetics* **6**: 423–428

Aoyama T, Yamano S, Guzelian PS, Gelboin HV and Gonzalez FJ (1990) Five of 12 forms of vaccinia virus-expressed human hepatic cytochrome P450 metabolically activate aflatoxin B1. *Proc Natl Acad Sci USA* **87**: 4790–4793

Badawi AF, Hirvonen A, Bell DA, Lang NP and Kadlubar FF (1995) Role of aromatic amine acetyltransferases, NAT1 and NAT2, in carcinogen-DNA adduct formation in the human urinary bladder. *Cancer Res* **55**: 5230–5237

Bergenheim US, Kunimi K, Collins VP and Ekman P (1991) Deletion mapping of chromosomes 8, 10 and 16 in human prostatic carcinoma. *Genes Chromosom Cancer* **3**: 215–220

Blum M, Grant DM, McBride W, Heim M and Meyer UA (1990) Human arylamine *N*-acetyltransferase genes: isolation, chromosomal localization, and functional expression. *DNA Cell Biol* **9**: 193–203

Bouchardy C, Benhamou S and Dayer P (1996) The effect of tobacco on lung cancer risk depends on CYP2D6 activity. *Cancer Res* **56**: 251–253

© Cancer Research Campaign 1998

Bulpitt CJ (1987) Confidence intervals. *Lancet* **1**: 494–497

Cannon L, Bishop DT, Skolnick M, Hunt S, Lyon JL and Smart CR (1982) Genetic epidemiology of prostate cancer in the Utah Mormon genealogy. *Cancer Surv* **1**: 47–69

Caporaso N and Goldstein A (1995) Cancer genes: single and susceptibility: exposing the difference. *Pharmacogenetics* **5**: 59–63

Caporaso N, Landi MT and Vineis P (1991) Relevance of metabolic polymorphisms to human carcinogenesis: evaluation of epidemiological evidence. *Pharmacogenetics* **1**: 4–19

Carter BS, Ewing CM, Ward WS, Treiger BF, Aalders TW, Schalken JA, Epstein JI and Isaacs WB (1990) Allelic loss of chromosomes 16q and 10q in human prostate cancer. *Proc Natl Acad Sci USA* **87**: 8751–8755

Carter BS, Beaty TH, Steinberg GD, Childs B and Walsh PC (1992) Mendelian inheritance of familial prostate cancer. *Proc Natl Acad Sci USA* **89**: 3367–3371

Catalona WJ, Smith DS, Ratliff TF and Basler JW (1993) Detection of organ-confined cancer is increased through prostate-specific antigen-based screening. JAMA **270**: 948–954

Chen ZR, Somogyi AA and Bochner F (1990) Simultaneous determination of dextromethorphan and three metabolites in plasma and urine using high-performance liquid chromatography with application to their disposition in man. *Ther Drug Monit* **12**: 97–104

Crespi CL, Penman BW, Gelboin HV and Gonzalez FJ (1991) A tobacco smoke-derived nitrosamine, 4(methylnitrosamino)-1-(3-pyridil)-1-butanone, is activated by multiple human cytochrome P450s including the polymorphic human cytochrome P4502D6. *Carcinogenesis* **12**: 1197–1201

Degawa M, Stern SJ, Martin MV, Guengerich FP, Fu PP, Ilett KF, Kaderlik RK and Kadlubar FF (1994) Metabolic activation and carcinogen-DNA adduct detection in human larynx. *Cancer Res* **54**: 4915–4919

Doll MA, Jiang W, Deitz AC, Rustan T and Hein DW (1997) Identification of a novel allele at the human NAT1 acetyltransferase locus. *Biochem Biophys Res Commun* **233**: 584–591

Evans DAP (1992) N-acetyltransferase. In *Pharmacogenetics of Drug Metabolism*, Kalow W (ed.), pp. 95–178. Pergamon Press: New York

Franke S, Klawitz I, Schnakenberg E, Rommel B, Van de Ven W, Bullerdiek J and Schloot W (1994) Isolation and mapping of a cosmid clone containing the human NAT2 gene. *Biochem Biophys Res Commun* **199**: 52–55

Feng Y, Jiang W and Hein DW (1996) 2-Aminofluorene-DNA adduct levels in tumor-target and nontarget organs of rapid and slow acetylator Syrian hamsters congenic at the NAT2 locus. *Toxicol Appl Pharmacol* **141**: 248–255

Gaedigk A, Blum M, Gaedigk R, Eichelbaum M and Meyer UA (1991) Deletion of the entire cytochrome P450 CYP2D6 gene as a cause of impaired drug metabolism in poor metabolizers of the debrisoquine/sparteine polymorphism. *Am J Hum Genet* **48**: 943–950

Gonzalez FJ, Skoda RC, Kimura S, Umeno M, Zanger UM, Nebert DW, Gelboin HV, Hardwick JP and Meyer UA (1988) Characterization of the common genetic defect in humans deficient in debrisoquin metabolism. *Nature* **331**: 442–446

Grant DM, Blum M, Beer M and Meyer UA (1990) Monomorphic and polymorphic human arylamine N-acetyltransferases: a comparison of liver isozymes and expressed products of two cloned genes. *Mol Pharmacol* **39**: 188–191

Grant DM, Hughes NC, Janezic SA, Goodfellow GH, Chen H, Gaedigk A, Yu VL and Grewal R (1997) Human acetyltransferase polymorphisms. *Mutat Res* **367**: 61–70

Guengerich FP and Shimada T (1991) Oxidation of toxic and carcinogenic chemicals by human cytochrome P-450 enzymes. *Chem Res Toxicol* **4**: 391–407

Heim M and Meyer UA (1991) Genetic polymorphism of debrisoquine oxidation: analysis of mutant alleles of CYP2D6 by restriction fragment analysis and by allele specific amplification. *Methods Enzymol* **206**: 173–183

Hein DW (1988) Acetylator genotype and arylamine-induced carcinogenesis. *Biochim Biophys Acta* **948**: 37–66

Hein DW, Rustan TD, Bucher KD, Furman EJ and Martin WJ (1991) Extrahepatic expression of the N-acetylation polymorphism towards arylamine carcinogens in tumor target organs of an inbred rat model. *J Pharmacol Exp Ther* **258**: 232–236

Hein DW, Doll MA, Rustan TD, Gray K, Feng Y, Ferguson RJ and Grant DM (1993) Metabolic activation and deactivation of arylamine carcinogens by recombinant human NAT1 and polymorphic NAT2 acetyltransferases. *Carcinogenesis* **14**: 1633–1638

Hirvonen A (1995) Genetic factors in individual responses to environmental exposures. *J Occup Environ Med* **37**: 37–43

Hubbard AL, Harrison DJ, Moyes C, Wyllie AH, Cunningham C, Mannion E and Smith CA (1997) N-acetyltransferase-2 genotype in colorectal cancer and selective gene retention in cancers with chromosome 8p deletions. *Gut* **41**: 229–234

Johansson I, Lundqvist E, Bertilsson L, Dahl ML, Sjoqvist F and Ingelman-Sundberg M (1993) Inherited amplification of an active gene in the cytochrome P450 CYP2D locus as a cause of ultrarapid metabolism of debrisoquine. *Proc Natl Sci USA* **90**: 11825–11829

Kadlubar FF and Badawi AF (1995) Genetic susceptibility and carcinogen-DNA adduct formation in urinary bladder carcinogenesis. *Toxicol Lett* **83**: 627–632

Kerry NL, Somogy AA, Bochner F and Mikus G (1994) The role of CYP2D6 in primary and secondary oxidative metabolism of dextromethorphan: in vitro studies using human liver microsomes. *Br J Clin Pharmacol* **38**: 243–248

Kloth MT, Gee RL, Messing EM and Swaminathan S (1994) Expression of N-acetyltransferase (NAT) in human uroepithelial cells. *Carcinogenesis* **15**: 2781–2787

Kunimi K, Bergerheim US, Larsson IL, Ekman P and Collins VP (1991) Allelotyping of human prostatic adenocarcinoma. *Genomics* **11**: 530–536

Mahler ER (1994) Genetics of urological cancers. *Br Med Bull* **50**: 698–707

Martínez C, Agúndez JAG, Olivera M, Martín R, Ladero JM and Benítez J (1995) Lung cancer and mutations at the polymorphic NAT2 gene locus. *Pharmacogenetics* **5**: 207–214

Moller-Jensen O, Esteve J, Moller H and Renard H (1990) Cancer in the European Community and its member states. *Eur J Cancer* **26**: 1167–1256

Murray GI, Taylor VE, McKay JA, Weaver RJ, Ewen SW, Melvin WT and Burke MD (1995) The immunohistochemical localization of drug-metabolizing enzymes in prostate cancer. *J Pathol* **177**: 147–152

Narod S, Dupont A, Cusan L, Diamond P, Gomez JL, Suburu R and Labrie F (1995) The impact of family history on early detection of prostate cancer. *Nature Med* **1**: 99–101

Neitzel HA (1986) A routine method for the establishment of permanent growing of lymphoblastoid cell lines. *Hum Genet* **73**: 320–322

Skoda RC, Gonzalez FJ, Demierre A and Meyer UA (1988) Two mutant alleles of the human cytochrome P450db1 gene (P450IID1) associated with genetically deficient metabolism of debrisoquine and other drugs. *Proc Natl Acad Sci USA* **85**: 5240–5243

Sone T, Zukowski K, Land SJ, King CM, Martin BM, Pohl LR and Wang CY (1994) Characteristics of a purified dog hepatic microsomal N,O-acyltransferase. *Carcinogenesis* **15**: 595–599

Tyndale RF, Aoyama T, Broly F, Matsunaga T, Inaba T, Kalow W, Gelboin HV, Meyer UA and Gonzalez FJ (1991) Identification of a new variant CYP2D6 allele lacking the codon encoding Lys-281: possible association with the poor metabolizer phenotype. *Pharmacogenetics* **1**: 26–32

Vatsis KP, Weber WW, Bell DA, Dupret JM, Evans DAP, Grant DM, Hein DW, Lin HJ, Meyer UA, Relling MV, Sim E, Suzuki T and Yamazoe Y (1995) Nomenclature for N-acetyltransferases. *Pharmacogenetics* **5**: 1–17

Vineis P, Bartsch H, Caporaso N, Harrington AM, Kadlubar FF, Landi MT, Malaveille C, Shields PG, Skipper P, Talaska G and Tannenbaum SR (1994) Genetically based N-acetyltransferase metabolic polymorphism and low-level environmental exposure to carcinogens. *Nature* **369**: 154–156

Yanagawa Y, Sawada M, Deguchi T, Gonzalez FJ, Kamataki T (1994) Stable expression of human CYP1A2 and N-acetyltransferases in Chinese hamster CHL cells: mutagenic activation of 2-amino-3-methylimidazo[4,5-f]quinoline and 2-amino-3,8-dimethylimidazo[4,5-f]quinoxaline. *Cancer Res* **54**: 3422–3427

© Cancer Research Campaign 1998